# Removal of methyl orange from water by Fenton oxidation of magnetic coconut-clothed biochar

**DOI:** 10.1039/d2ra03545f

**Published:** 2022-08-30

**Authors:** Jia Xu, Qianhui Ma, Wen Feng, Xiaopeng Zhang, Qiang Lin, Chenghang You, Xianghui Wang

**Affiliations:** Key Laboratory of Water Pollution Treatment and Resource Reuse of Hainan Province, Key Laboratory of Soil Pollution Remediation and Resource Reuse of Haikou City, College of Chemistry and Chemical Engineering, Hainan Normal University Haikou 571158 China youchh@163.com god820403@163.com

## Abstract

Water pollution has become a serious environmental problem to date. Advanced oxidation processes (AOP) have been widely applied in water treatments. However, the traditional Fenton reaction based on the Fe^2+^–H_2_O_2_ system has obvious drawbacks, limiting further practical applications. In this work, an Fe_3_O_4_ and nano-clothed biochar (Fe_3_O_4_/CBc) composite was prepared through a precipitation method and used for the degradation of methyl orange (MO) in water. The Fe_3_O_4_/CBc composite was characterized by FTIR, BET, SEM, TEM, XRD, and VSM. In addition, the adsorption/catalytic oxidation of MO were also tested. Specifically, Fe_3_O_4_/CBc had a rough surface, abundant porous structure, high surface area of 835.82 m^2^ g^−1^, and obvious magnetization. The catalyst showed rather high performance towards MO removal. The optimal conditions for MO removal were as follows: the dosage of hydrogen peroxide was 16 mmol L^−1^, pH = 3, the temperature was 35 °C, and the addition amount of adsorbent was 10 mg. Under optimal conditions, the MO removal rate can be higher than 99%. The synergistic effect between catalytic degradation and adsorption in removing MO was also observed. Besides high performance in removing MO, Fe_3_O_4_/CBc also exhibited high stability, easy magnetic separation, and great reusability, as well as the potential to be developed as a new heterogeneous Fenton catalyst.

## Abstract 2

Water pollution has become a serious environmental problem to date. Advanced oxidation processes (AOP) have been widely applied in water treatments.

## Graphical abstract

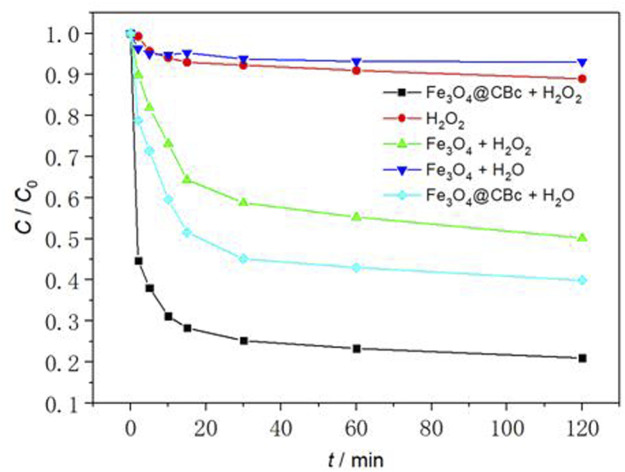

## Introduction

With the continuous development of modern industry, water pollution is becoming more and more serious. For example, the textile industry is one of the most important industries. There are many production processes in this industry, which have produced highly polluted wastewater containing dyes with different structures. If the treatment method is improper, this kind of wastewater has color and toxicity, which will cause serious environmental problems. Therefore, it is urgent to develop efficient approaches to control water pollution. As compared with other advanced oxidation processes, the Fenton reaction^1^ is an advanced oxidation method with the advantages of mild conditions, low cost, and high efficiency. It can oxidize and degrade almost all kinds of organic pollutants without selectivity.^2,3^ Therefore, it has been widely used in wastewater treatments in areas like papermaking, printing, dyeing, *etc.* Unfortunately, the traditional Fenton reaction^4–6^ usually suffers from several defects including difficult reuse of catalyst, low utilization of H_2_O_2,_ and easy production of iron sludge. Thus, it would be of great importance to improve the Fenton system^7^ in water treatment.

Fe_3_O_4_ NPs have the characteristics of super-paramagnetism, great thermal stability, high reaction activity, and simple preparation.^8–10^ They can replace Fe^2+^ in the traditional homogeneous Fenton reaction and stimulate H_2_O_2_ to produce ˙OH as a new Fenton reaction catalyst. It not only retains the characteristics of high catalytic activity of Fenton reaction, but also utilizes magnetic separation with efficient recovery, which might overcome the defects of homogeneous Fenton reactions and attract extensive attention. In this work, a super-paramagnetic Fenton catalyst (Fe_3_O_4_/CBc) was fabricated by loading Fe_3_O_4_ NPs on coconut-clothed biochar^11,12^ through an *in situ* precipitation procedure.^13^ Methyl orange (MO) was selected as the target pollutant to evaluate the degradation performance of organic contaminants. The effects of addition amount, H_2_O_2_ content, pH, temperature, and free radical inhibitor on MO removal were also studied.^14^

## Experimental

### Reagents

Self-made coconut-clothed (coconut-clothed was obtained from the discarded coconuts after eating). KOH, HCL, Methyl orange (MO), FeCl_3_·6H_2_O, FeCl_2_·7H_2_O, 30% H_2_O_2_ and C_2_H_5_OH were ordered from Sinopharm Chemical Reagent Co., Ltd All reagents were analytical pure grade.

#### Preparation of modified coconut-clothed biochar (CBc)

Coconut-clothed material (5.0 g), KOH (15.0 g), and deionized water (50 mL) were placed in a 100 mL glass beaker. The mixture was stirred for 2 h, which was then placed into an oven at 80 °C for drying. The dried sample was placed in a quartz boat and pyrolyzed at 700 °C for 270 min with a heating rate of 10 °C min^−1^ under the protection of pure nitrogen. After cooling down naturally, the sample obtained was washed to neutral and dried at 70 °C under the vacuum.

#### Preparation for Fe_3_O_4_/CBc

Deionized water (300 mL) was added into a 500 mL three-port flask equipped with a mechanical stirring device. Nitrogen was purged into the flask for 30 min to remove the air inside the flask. Then FeCl_3_·6H_2_O (4.49 g, 16.61 mmol) and FeSO_4_·7H_2_O (2.31 g, 8.30 mmol) were added and stirred for 20 min to obtain a homogeneous orange-red solution. Then CBc (1.9 g, the mass ratio of CBc and Fe_3_O_4_ is 1 : 1) was added and stirred for another 3 h before adjusting the pH to about 10 using NH_3_·H_2_O. The obtained black suspension was aged at 60 °C for 4 h, followed by cooling down naturally, separating by a magnet, washing with deionized water five times and C_2_H_5_OH three times, and drying at 60 °C under the vacuum over 20 h. Finally, the biomass carbon composite was obtained and named as Fe_3_O_4_/CBc. Samples with other proportions (m(CBc) : m(Fe_3_O_4_) = 3 : 1, 2 : 1) were also prepared through the same above experimental steps.

### MO Removal experiments

Methyl orange solution (25 mL, 100 mg L^−1^) was added into a 50 mL conical flask to simulate wastewater. The pH of the solution was adjusted with 0.1 mol L^−1^ hydrochloric acid or sodium hydroxide. After adding a certain mass of H_2_O_2_ and Fe_3_O_4_/CBc, the mixture was vibrated for 120 min under room temperature. After that, the concentration of methyl orange was measured using visible spectrophotometry.

### Characterizations

Brunauer–Emmett–Teller (ASAP2460) was used to measure the nitrogen adsorption capacity of samples at 77 K and the specific surface area of each sample. Scanning electron microscopy (JSM-7401F) and Transmission electron microscopy (JEM-F200) were employed to record the micro surface morphology of materials. Fourier transform infrared spectroscopy (FT-IR6700) was used for further infrared spectrum analysis of materials. X-ray diffraction (UItima IV) was utilized to test the crystal structure of materials. Vibrating sample magnetometer (SQUID-VSM) was used to test the hysteresis loop of catalyst. The concentration change of methyl orange solution was analyzed by a ultraviolet-visible spectrophotometer (UV, 752N).

## Results and discussion

### SEM, TEM, and EDS analysis

**Fig. 1:**
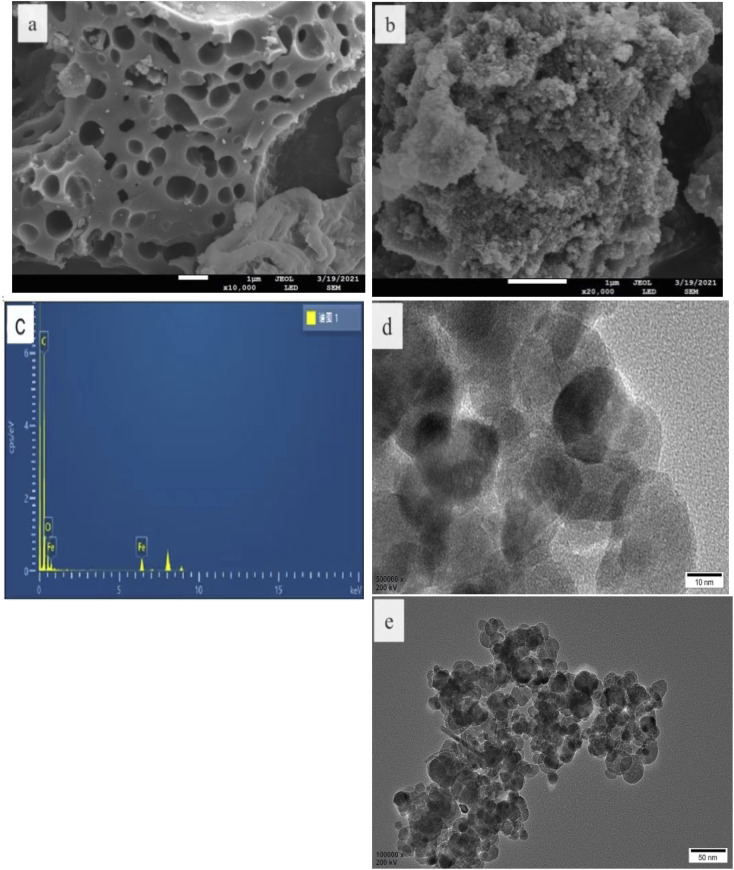
SEM, TEM, EDS, and HRTEM of CBc and Fe_3_O_4_/CBc. (a) CBc SEM. (b) Fe_3_O_4_/CBc SEM. (c) Fe_3_O_4_/CBc EDS. (d) Fe_3_O_4_/CBc TEM. (e) Fe_3_O_4_/CBc HRTEM.

The morphology and structure of the as-prepared catalysts were characterized by SEM and TEM. As shown in Fig. 1a and d, the CBc had a rather rough surface with numerous porous structures,^15,16^ and the Fe_3_O_4_ nanoparticles had an average diameter of about 50 nm and the surface was rough, which can be attributed to the fact that each Fe_3_O_4_ nanospheres are composed of many smaller particles. The EDS spectrum in Fig. 1c showed that the composite contains C, O, and Fe. As seen in Fig. 1d, the particle size of Fe_3_O_4_ was about 50 nm. From the HRTEM image (Fig. 1e) of the Fe_3_O_4_/CBc^17^ diagram of the composite, the lattice stripes were 0.292 and 0.249 nm, respectively, which were corresponding to the 220 and 311 facets of Fe_3_O_4_ respectively, confirming that these NPs were Fe_3_O_4_.

### BET analysis

**Fig. 2:**
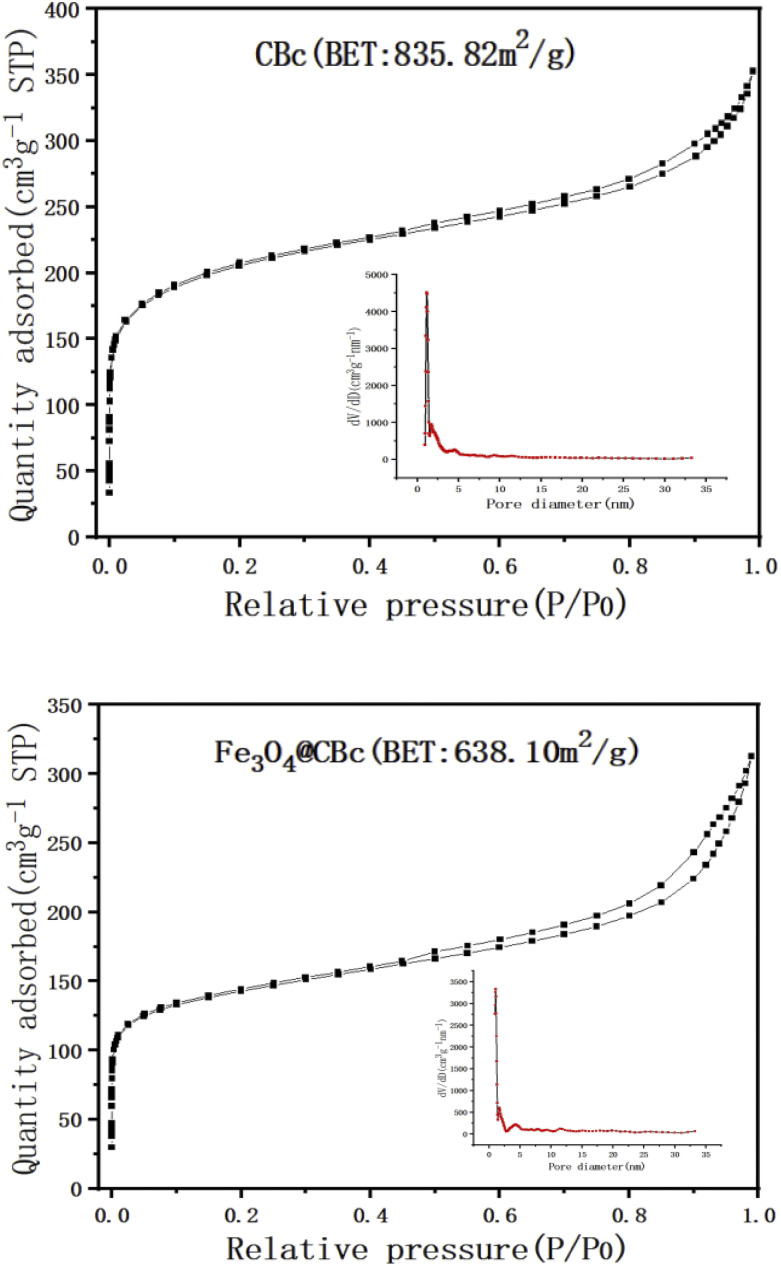
Nitrogen adsorption and desorption curve and pore size distribution of CBc and Fe_3_O_4_/CBc.

The surface area and the porosity of the CBc and Fe_3_O_4_/CBc were summarized in Fig. 2. In Fig. 2, the surface area of the CBc was higher as compared to the surface area of the Fe_3_O_4_/CBc. The immobilization of the Fe_3_O_4_ on the biochar surface didn't provide the additional surface area for adsorption, which is possible due to the particles clogged the pores in the surface of the biochar. In fact, the pore volume of the Fe_3_O_4_/CBc was slightly lower as compared to that of the CBc, which changed from 0.475 cm^3^ g^−1^ to 0.411 cm^3^ g^−1^. It indicated that the introduction of Fe_3_O_4_ nanoparticles can make the surface of the material rough, in which the nanoparticles were overlapped and piled up with each other, resulting in the reduction of the empty volume and the decrease of the specific surface area.

### FT-IR analysis

**Fig. 3:**
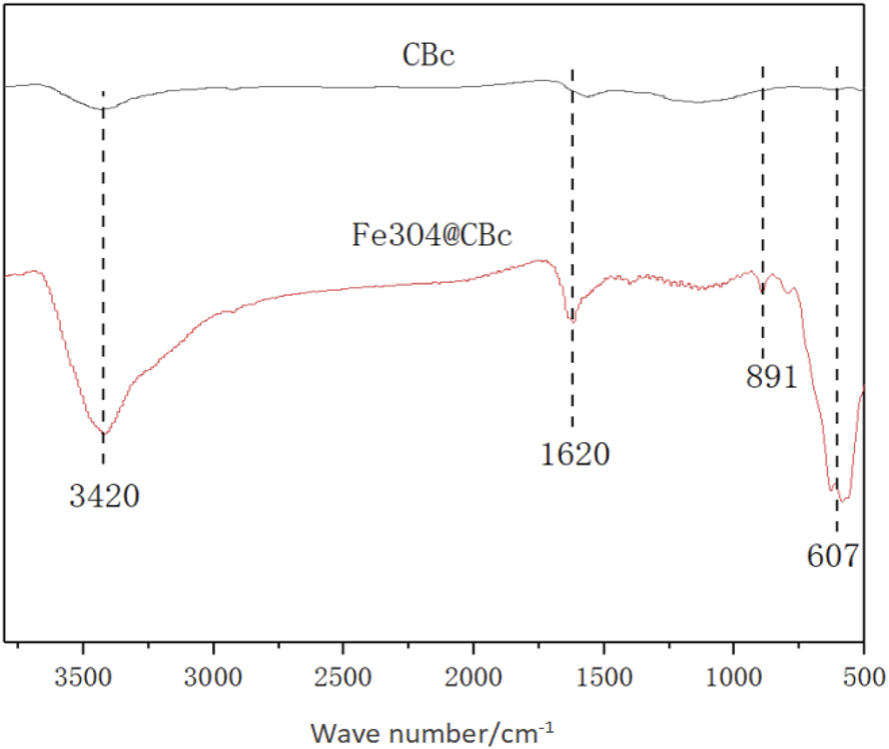
FT-IR spectra of CBc and Fe_3_O_4_/CBc.

The IR spectra of the CBc and Fe_3_O_4_/CBc were shown in Fig. 3. CBc and Fe_3_O_4_/CBc composite had roughly the same absorption peaks. The adsorption peaks around 3420, 891, and 1620 cm^−1^ can be assigned to the stretching vibration peaks of O–H, C=C, and C=O, respectively, indicating that the surface of the composite was rich in oxygen-containing functional groups, which was conducive to the adsorption of organic compounds. Specifically, Fe_3_O_4_/CBc showed an absorption peak at 607 cm^−1^ in the infrared image, which was the bending vibration peak of Fe–O–Fe,^18,19^ suggesting that Fe_3_O_4_ was successfully loaded on the surface of coconut-clothed biochar.

### XRD analysis

**Fig. 4:**
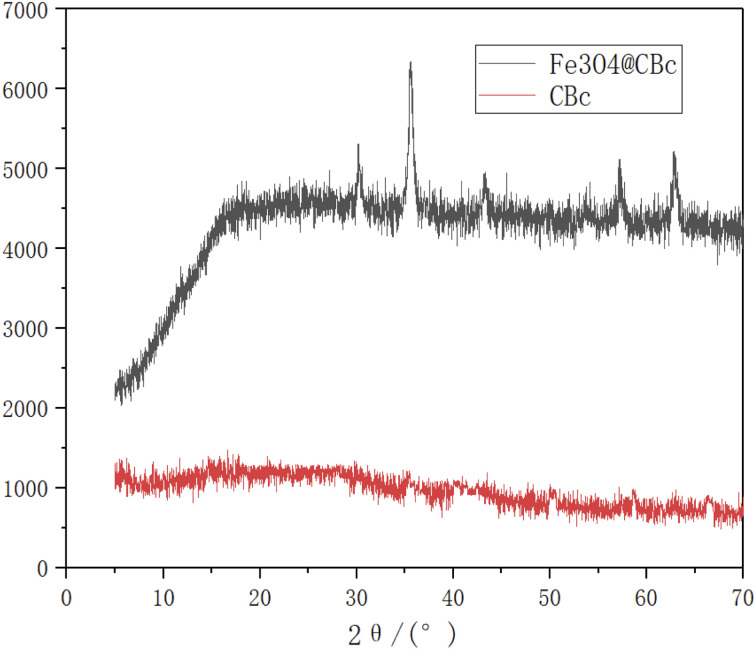
XRD patterns of CBc and Fe_3_O_4_/CBc.

As shown in Fig. 4, the Fe_3_O_4_/CBc composite had five diffraction peaks at 30.56, 35.58, 43.32, 57.2, and 62.84°, which were consistent with the characteristic peaks of Fe_3_O_4_.

### VSM analysis

**Fig. 5:**
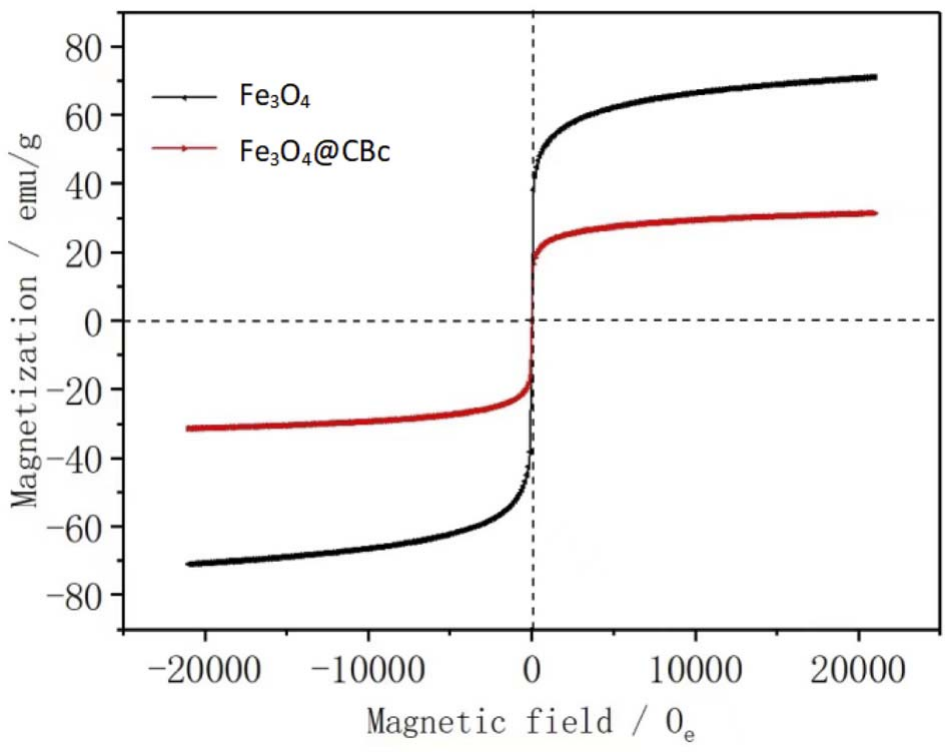
Magnetization curves of CBc and Fe_3_O_4_/CBc composites.

Fig. 5 showed the hysteresis loops of Fe_3_O_4_ and Fe_3_O_4_/CBc. It can be found that Fe_3_O_4_/CBc had a saturation magnetization of 23.4 emu g^−1^, *vs.* 69.48 emu g^−1^ for Fe_3_O_4_. There was no hysteresis phenomenon. Furthermore, the values of residual coercivity and magnetization were zero, which reflects that the target materials were superparamagnetic.^20^ Although the saturation magnetization of the composite was less than that of Fe_3_O_4_, the value was still higher than 16.3 emu g^−1^,^21^ which was required for general magnetic separations. Therefore, Fe_3_O_4_/CBc can be easily separated through the external magnetic field, which can be further confirmed by the rapid magnetic separation of the catalyst.

### Removal effect of MO under different conditions

**Fig. 6:**
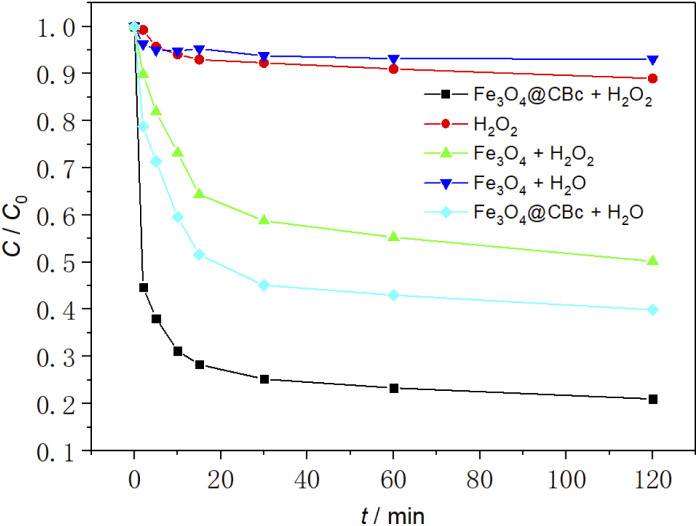
Effect of different conditions on MO removal.

As shown in Fig. 6, the removal effect of MO was studied under different conditions. The initial temperature was 25 °C, the initial pH was 3.0, the amount of catalyst was 5 mg and the concentration of 30% H_2_O_2_was 16 mmol L^−1^.

The experimental results showed that when sole H_2_O_2_ was added, the MO removal rate was only 10.3%. This was because H_2_O_2_ without the presence of a catalyst produces ˙OH slowly and cannot achieve rapid oxidative degradation of MO. When Fe_3_O_4_ was used alone, since Fe_3_O_4_ cannot produce ˙OH, MO was removed only by the adsorption of Fe_3_O_4_. As a reasonable result, the MO removal rate was very low (6.1%). For Fe_3_O_4_/CBc, it can be found that it can exhibit a MO removal rate of 59.8%, much higher than those using sole CBc or Fe_3_O_4_, suggesting that there might exist a synergistic effect between CBc and Fe_3_O_4_. In the catalyst/hydrogen peroxide system, the catalytic degradation rate of MO by Fe_3_O_4_/CBc was much higher than that using Fe_3_O_4_ alone, and the MO removal rate was as high as 78.9% in the first 45 min, much higher than those of adding H_2_O_2_ and Fe_3_O_4_ + H_2_O groups. This result should be attributed to the more ˙OH due to the interaction between Fe_3_O_4_ and H_2_O_2_, which can easily attack the MO adsorbed on the surface of CBc. Therefore, the efficient removal of MO by Fe_3_O_4_/CBc and hydrogen peroxide system was resulted from the synergistic effect of efficient catalytic oxidation and CBc adsorption.

### Effect of the amount of Fe_3_O_4_/CBc and H_2_O_2_ on the degradation of MO

**Fig. 7:**
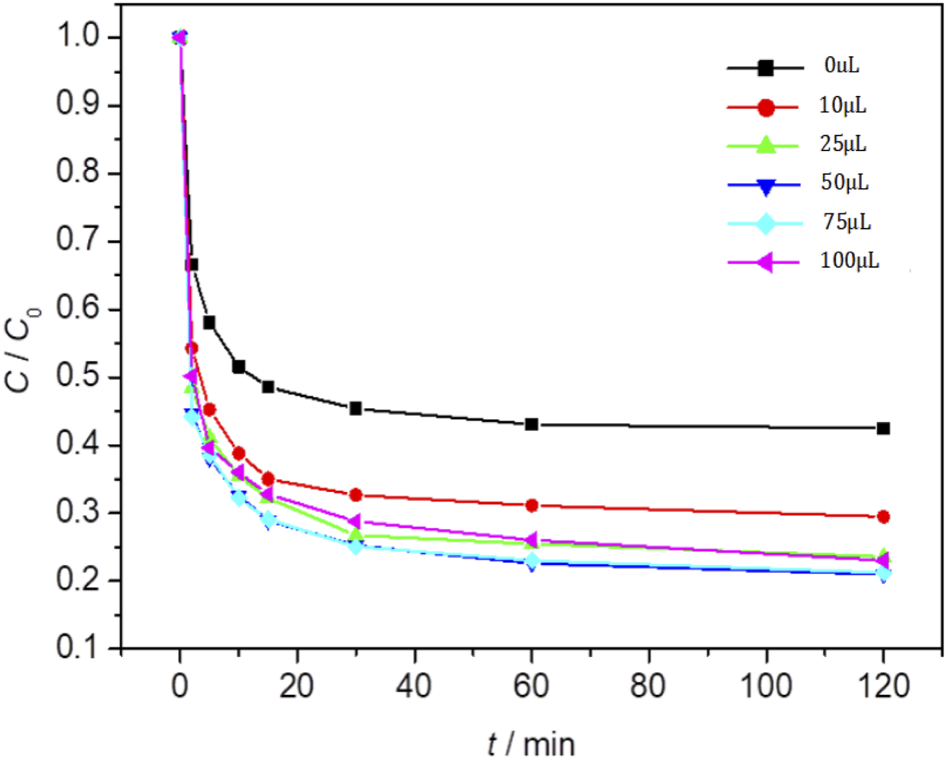
Fe_3_O_4_/CBc Effect of addition amount on oxidation and degradation of MO.

**Fig. 8:**
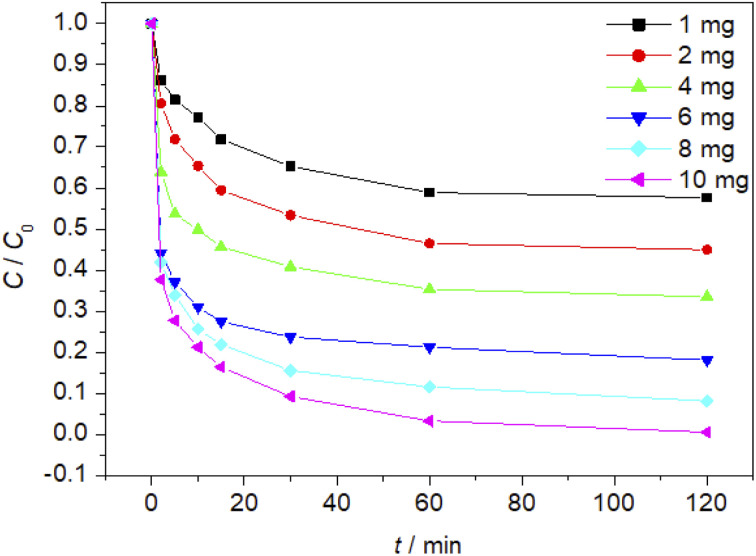
Effect of hydrogen peroxide addition on MO oxidation and degradation process.

The effect of Fe_3_O_4_/CBc and H_2_O_2_ usages on the MO removal were shown in Fig. 7 and 8, respectively. The MO removal rate increased from 41.9% to 99.4% as Fe_3_O_4_/CBc usage raised from 1 mg to 10 mg. In addition, the removal rate of MO was enhanced with the increase of Fe_3_O_4_/CBc amount. When the addition amount of catalyst was fixed to be 5.0 mg, the MO removal rate increased from 70.2% to 76.8% as the hydrogen peroxide usage increased from 10 to 100 μL. When the addition amount of hydrogen peroxide exceeds 16 mmol L^−1^, the removal rate of MO also decreased slightly.

According to research reports, we proposed the possible mechanism of the Fenton reaction *as follows*:

1 Fe^2+^ + H_2_O_2_ → Fe^3+^ + OH^−^ + ˙OH

2 Fe^3+^ + H_2_O_2_ → Fe^2+^ + H^+^ + ˙O_2_H

3 Fe^2+^ + ˙OH → Fe^3+^ + OH^−^

4 Fe^3+^ + ˙O_2_H → Fe^2+^ + H^+^ + O_2_

5 H_2_O_2_·+ ˙OH → H_2_O·+ ˙O_2_H

From the reaction mechanism, when the amount of Fe_3_O_4_/CBc and H_2_O_2_ is sufficient,^22^ the Fe^2+^ reacted sufficiently with H_2_O_2_ to produce hydroxyl radicals. When the amount of Fe_3_O_4_/CBc and hydrogen peroxide was gradually increased, the formed ˙OH increases, which can improve MO removal;^23^ However, when the addition amount of Fe_3_O_4_/CBc or H_2_O_2_ exceeded the critical value, excess ˙OH would act with H_2_O_2_ to quench the free radical reaction, which was not conducive to the oxidative degradation of MO.

### Effect of initial pH value on MO degradation process

**Fig. 9:**
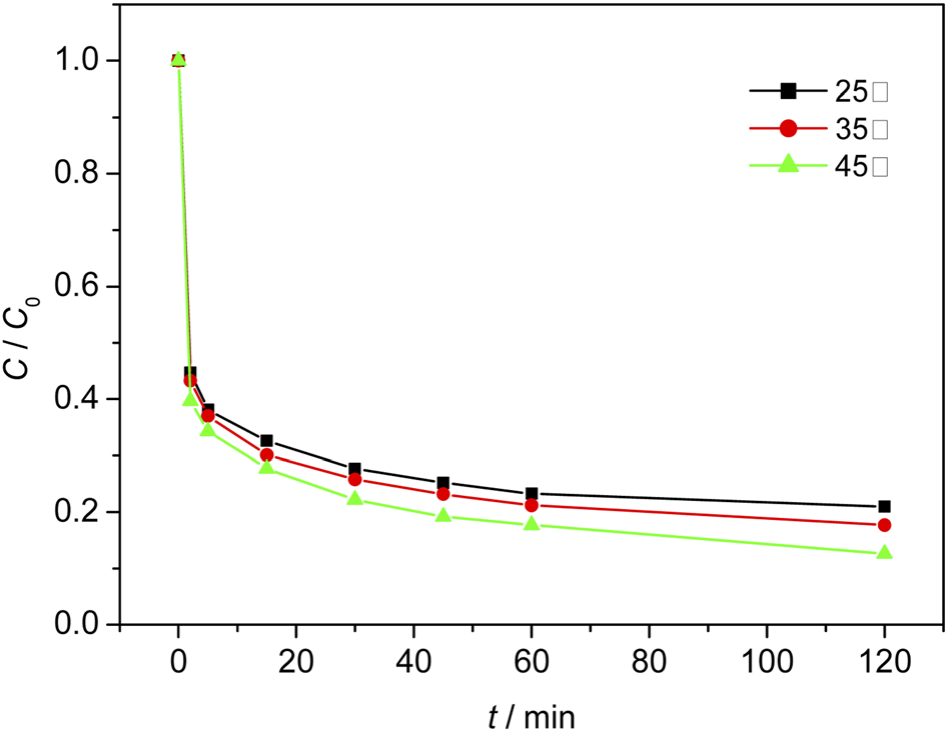
Effect of initial pH value on MO degradation.

At an ambient temperature of 25 °C, the dosage of Fe_3_O_4_/CBc was 5 mg and the concentration of 30% hydrogen peroxide was 15.99 mmol L^−1^. The influence of the solution pH values on the MO degradation was also studied, as is shown in Fig. 8. As seen from Fig. 9a that the pH of the solution has a great influence. With the pH value between 5 and 8, the removal efficiency was low, which should be attributed to the formation of hydrate iron and hydrated ferrous complexes. Since the divalent iron ions cannot be effectively dissociated, the generation of ˙OH was inhibited, resulting in the obvious weakening of the oxidative degradation ability of Fenton system.^24^ In this case, the removal of MO mainly depended on the adsorption of catalyst materials. When the pH of the solution was between 2 and 4, there was electrostatic adsorption between Fe_3_O_4_/CBc and MO, which increases the adsorption capacity of MO at lower pH, as well as the contact of active sites, and eventually improves the degradation rate. When the pH value was 4, the MO removal rate was 53.3%, and when the pH value was 3, the MO removal rate reached up to 78.6%. So the best removal rate can be obtained with the pH at 3.

In Fig. 9b, when the pH values were 4, 3 and 2, the dissolution amount of Fe were 0.34 mg L^−1^, 3.89 mg L^−1^ and 12.6 mg L^−1^ respectively. The dissolution amount of Fe increased as the pH decreases. The change of Fe dissolution amount can directly reflect the relationship between MO removal rate and pH value. Finally, Fe^2+^ produced in Fenton system reached up to the critical value when the pH value was 3;^25^ When the pH value was 2, because the dissolution of Fe exceeded the adjacent value, the free radical quenching reaction between H_2_O_2_ and ˙OH occurred, resulting in the decrease of the concentration of hydrogen peroxide and ˙OH in the system. At the same time, due to the synergistic effect of adsorption, the MO removal rate did not significantly change. Considering the environmental impact and the analysis of the experimental results, the optimal pH value in the experiment was 3.

### Effect of ambient temperature on the degradation process of MO

**Fig. 10:**
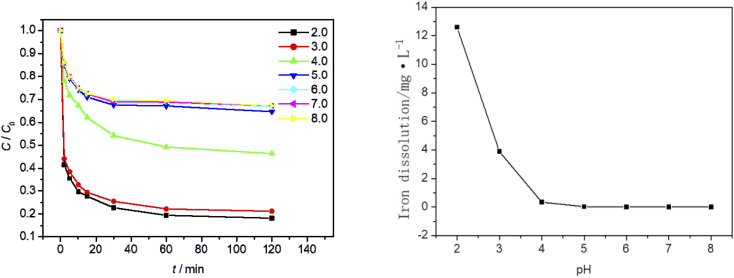
Effect of reaction temperature on MO removal efficiency.

When the initial pH of the solution was 3, the dosage of Fe_3_O_4_/CBc was 2 mg and the dosage of H_2_O_2_ was 16 mmol L^−1^. The effect of temperature on the MO removal process was shown in Fig. 10. The experimental results showed that at 25 °C, 35 °C, and 45 °C, the removal rates of MO were 78.9%, 82.2%, and 87.3% respectively. It was proved that a higher temperature can facilitate the removal of MO due to the higher dissolution of Fe and the faster reaction rates at a higher temperature. However, if the temperature was too high, the decomposition rate of hydrogen peroxide was also accelerated and additional facilities or equipment were required to maintain the reaction temperature, resulting in a large amount of energy consumption. Therefore, based on the cost assessment, the reaction was carried out at room temperature. Since the heat energy can be improved in combination with the production process, 35 °C would be the best treatment temperature.

### Effect of inhibitors on MO degradation

**Fig. 11:**
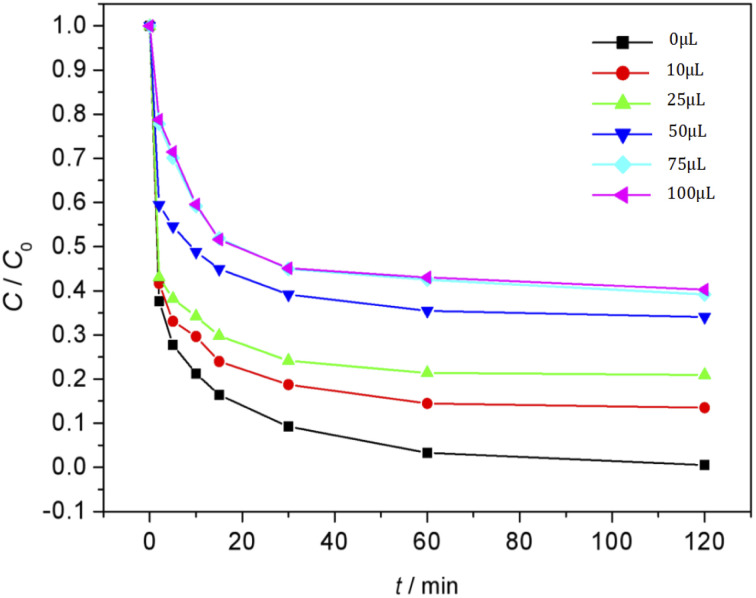
Effect of *t*-butanol inhibitor on MO removal.

MO degradation reaction was carried out by the Fenton reaction,^26,27^ which depends on the strong oxidizing free radical ˙OH produced by the system. Therefore, the free radical scavenger in the reaction system would have a very direct impact on the degradation process of MO. In the experiment, the dosage of Fe_3_O_4_/CBc was 5 mg and the dosage of H_2_O_2_ was 16 mmol L^−1^. When the initial pH of the solution was 3 and the temperature was 25 °C, *t*-butanol was used as a free radical scavenger to explore the relationship between the removal effect of MO and the addition amount of *t*-butanol. The experimental results were shown in Fig. 11. There was a close relationship between the MO removal rates and the additional amounts of *tert* butyl. With only 10 μL *t*-butanol addition, the MO removal rate decreased drastically from 99.6% to 86.3%. When the *t*-butanol usage reached up to 39 mmol L^−1^, the MO^28^ removal rate decreased to only 60.5%. When the addition amount of *t*-butanol continued to increase up to 78 mmol L^−1^, the MO removal rate was no longer changed. According to the experimental data, when the amount of *t*-butanol was more than 39 mmol L^−1^, the Fenton system was almost completely inhibited, resulting in the complete stop of oxidative degradation, the MO removal mainly depended on the adsorption of MO by Fe_3_O_4_/CBc (Fig. 7, the adsorption of MO on Fe_3_O_4_/CBc was 59.5% without H_2_O_2_). Therefore, the existence of free radical inhibitors like *t*-butanol should be avoided in the Fenton reaction system.

### Catalyst regeneration performance

**Fig. 12:**
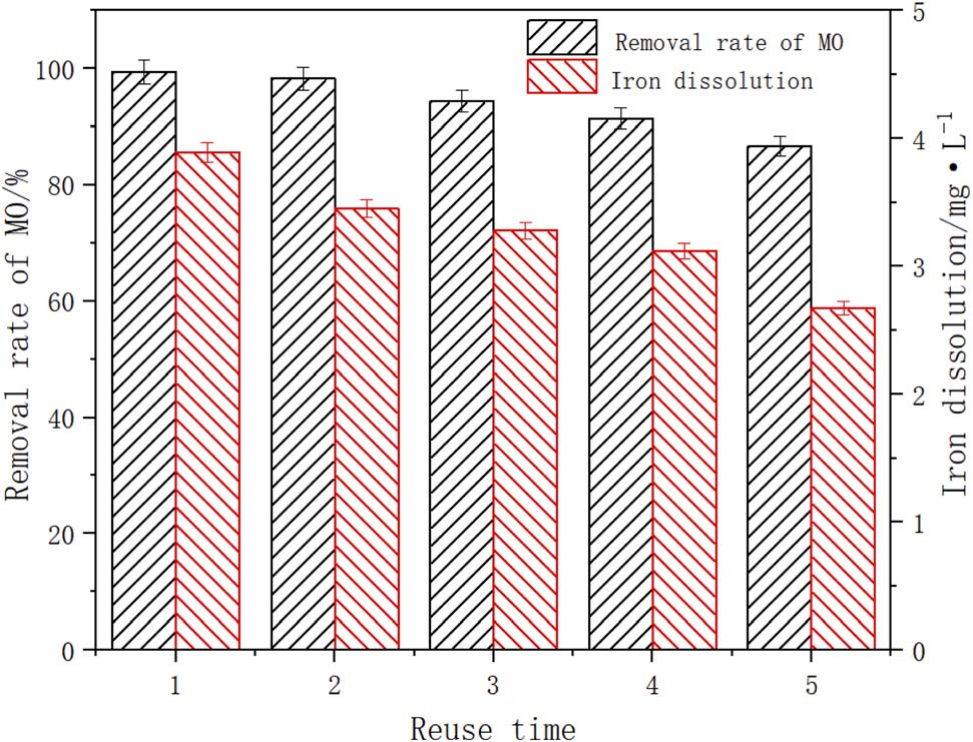
The effect of catalyst reuse on MO removal and the dissolution of Fe.

When the initial pH of the solution was 3, the ambient temperature was 25 °C, the dosage of Fe_3_O_4_/CBc was 10 mg and the dosage of H_2_O_2_ was 16 mmol L^−1^ to test the reuse efficiency of the catalyst. After the experiment, the Fe_3_O_4_/CBc was separated by magnetic separation, washed with deionized water and microwaved for 1 h, and reuse 4 times. The experimental data were shown in Fig. 12. After the catalyst was utilized five times, the MO removal rates showed a downward trend, and the removal rates of MO were 99.4%, 98.2%, 94.3%, and 86.7% respectively. Although the removal efficiency slightly decreased, it was still higher than 85%, indicating that it had a great reuse rate. There might be two reasons for the decline of the MO removal effect. On the one hand, there was a certain loss of Fe_3_O_4_ in the degradation process. The dissolution amount of Fe decreased from 3.89 to 2.67 mg L^−1^ with the increase in catalyst use times, resulting in the decline of catalytic efficiency. On the other hand, some MO molecules were firmly adsorbed on the material surface, which occupied the adsorbed active sites and cannot be effectively removed by activation. In a comprehensive analysis, Fe_3_O_4_/CBc was stable,^29,30^ easily separately, and had a high reuse efficiency.

## Conclusion

In summary, a Fe_3_O_4_ and coconut-clothed biochar (Fe_3_O_4_/CBc) composite is prepared through a precipitation method and employed for the degradation of methyl orange (MO) in water. The Fe_3_O_4_/CBc composite was characterized by FTIR, BET, SEM, TEM, XRD, and VSM. The adsorption and catalytic oxidation of MO were also tested. Specifically, Fe_3_O_4_/CBc has a rough surface, abundant porous structures, a high surface area of 638.10 m^2^ g^−1^, and obvious magnetization. The catalyst showed rather high performance towards MO removal. The optimal conditions for MO removal were obtained as follows: the dosage of hydrogen peroxide was 16 mmol L^−1^, pH = 3, the temperature was 35 °C, and the addition amount of adsorbent was 10 mg. Under optimal conditions, the MO removal rate can be higher than 99.4%. The synergistic effect between catalytic degradation and adsorption in removing MO was also discovered. Besides high performance in the depredating MO, Fe_3_O_4_/CBc also exhibited high stability, easy magnetic separation, and good reusability, as well as the potential to be developed as a new heterogeneous Fenton catalyst.

## Conflicts of interest

There are no conflicts to declare.

## Supplementary Material

## References

[cit1] Giwa A.-R. A., Bello I. A., Olabintan A. B. (2020). *et al.*, Kinetic and thermodynamic studies of fenton oxidative decolorization of methylene blue. Heliyon.

[cit2] Usman M., Ho Y.-S. (2020). A bibliometric study of the Fenton oxidation for soil and water remediation. J. Environ. Manage..

[cit3] Hu R. (2020). Pollution control and remediation of rural water resource based on urbanization perspective. Environ. Technol. Innovation.

[cit4] Unal B. O., Bilici Z., Ugur N. (2019). *et al.*, Adsorption and Fenton oxidation of azo dyes by magnetite nanoparticles deposited on a glass substrate. J. Water Proc. Eng..

[cit5] Zhang J., Zhang G., Ji Q. (2020). *et al.*, Carbon nanodot-modified FeOCl for photo-assisted Fenton reaction featuring synergistic in-situ H_2_O_2_ production and activation. Appl. Catal., B.

[cit6] Yoon S., Bae S. (2018). Novel synthesis of nanoscale zerovalent iron from coal fly ash and its application in oxidative degradation of methyl orange by Fenton reaction. J. Hazard. Mater..

[cit7] Shen L., Wang W., Li T. (2019). *et al.*, Powdered activated coke for COD removal in the advanced treatment of mixed chemical wastewaters and regeneration by Fenton oxidation. Chem. Eng. J..

[cit8] Bi J., Huang X., Wang J. (2019). *et al.*, Oil-phase cyclic magnetic adsorption to synthesize Fe_3_O_4_@C@TiO_2_-nanotube composites for simultaneous removal of Pb(II) and Rhodamine B. Chem. Eng. J..

[cit9] Zhang Q., Zhang W., Peng K. (2019). In-situ synthesis and magnetic properties of core-shell structured Fe/Fe_3_O_4_ composites. J. Magn. Magn. Mater..

[cit10] Qian L., Peng J., Xiang Z. (2018). *et al.*, Effect of annealing on magnetic properties of Fe/Fe_3_O_4_ soft magnetic composites prepared by in-situ oxidation and hydrogen reduction methods. J. Alloys Compd..

[cit11] Yi Y., Huang Z., Lu B. (2020). *et al.*, Magnetic biochar for environmental remediation: A review. Bioresour. Technol..

[cit12] Hao Z., Wang C., Yan Z. (2018). *et al.*, Magnetic particles modification of coconut shell-derived activated carbon and biochar for effective removal of phenol from water. Chemosphere.

[cit13] Li Z., Chen Z., Zhu Q. (2020). *et al.*, Improved performance of immobilized laccase on Fe_3_O_4_@C-Cu^2+^ nanoparticles and its application for biodegradation of dyes. J. Hazard. Mater..

[cit14] Godwin Patrick M., Pan Y., Xiao H. (2019). *et al.*, Progress in Preparation and Application of Modified Biochar for Improving Heavy Metal Ion Removal From Wastewater. J. Bioresour. Bioprod..

[cit15] Park J.-H., Wang J. J., Meng Y. (2019). *et al.*, Adsorption/desorption behavior of cationic and anionic dyes by biochars prepared at normal and high pyrolysis temperatures. Colloids Surf., A.

[cit16] Chen S., Qin C., Wang T. (2019). *et al.*, Study on the adsorption of dyestuffs with different properties by sludge-rice husk biochar: Adsorption capacity, isotherm, kinetic, thermodynamics and mechanism. J. Mol. Liq..

[cit17] Wu L., Wei C., Zhang S. (2019). *et al.*, MgO-modified biochar increases phosphate retention and rice yields in saline-alkaline soil. J. Cleaner Prod..

[cit18] Qiu Y., Xu X., Xu Z. (2020). *et al.*, Contribution of different iron species in the iron-biochar composites to sorption and degradation of two dyes with varying properties. Chem. Eng. J..

[cit19] Xu S., Li J., Yin Z. (2020). *et al.*, A highly efficient strategy for enhancing the adsorptive and magnetic capabilities of biochar using Fenton oxidation. Bioresour. Technol..

[cit20] Cai W., Wei J., Li Z. (2018). *et al.*, Preparation of amino-functionalized magnetic biochar with excellent adsorption performance for Cr(VI) by a mild one-step hydrothermal method from peanut hull. Colloids Surf., A.

[cit21] Premarathna K. S. D., Rajapaksha A. U., Adassoriya N. (2019). *et al.*, Clay-biochar composites for sorptive removal of tetracycline antibiotic in aqueous media. J. Environ. Manage..

[cit22] Liu S., Li J., Xu S. (2019). *et al.*, A modified method for enhancing adsorption capability of banana pseudostem biochar towards methylene blue at low temperature. Bioresour. Technol..

[cit23] Schwarze M., Seo D., Bibouche B. (2020). *et al.*, Comparison of positively charged polymer species and cationic surfactants for methyl orange removal via polyelectrolyte and micellar enhanced ultrafiltration. J. Water Proc. Eng..

[cit24] Xiao C., Li H., Zhao Y. (2020). *et al.*, Green synthesis of iron nanoparticle by tea extract (polyphenols) and its selective removal of cationic dyes. J. Environ. Manage..

[cit25] Babuponnusami A., Muthukumar K. (2014). A review on Fenton and improvements to the Fenton process for wastewater treatment. J. Chem. Environ. Eng..

[cit26] Yu Y., Huang F., He Y., Liu X. (2019). *et al.*, Heterogeneous Fenton-like degradation of ofloxacin over sludge derived carbon as catalysts: Mechanism and performance. Sci. Total Environ..

[cit27] García-Leiva B., Teixeira L. A. C., Torem M. L. (2019). Degradation of xanthate in waters by hydrogen peroxide, Fenton and simulated solar photo-Fenton processes. J. Mater. Res. Technol..

[cit28] Meili L., Lins P. V., Zanta C. L. P. S. (2019). *et al.*, MgAl-LDH/Biochar composites for methylene blue removal by adsorption. Appl. Clay Sci..

[cit29] Wang J., Ma Q., Zhang Z. (2020). *et al.*, Bacteria mediated Fenton-like reaction drives the biotransformation of carbon nanomaterials. Sci. Total Environ..

[cit30] Shang H., Wang Q., Ok Y. S. (2021). *et al.*, Magnetic biochar production alters the molecular characteristics and biological response of pyrolysis volatile-derived water-soluble organic matter. Sci. Total Environ..

